# Multiple simple cystic metastases in the lateral neck at presentation with papillary thyroid microcarcinoma: A case report

**DOI:** 10.1097/MD.0000000000023866

**Published:** 2021-01-29

**Authors:** Lei Zhu, Xi Zhu, Bin Zhou, Wei bo Mao, Yong Wu, Feng Cheng

**Affiliations:** a Department of Thyroid and Breast Surgery, Lishui Hospital of Zhejiang University, Lishui Municipal Central Hospital, Lishui; b Department of Pathology, Lishui Hospital of Zhejiang University, Lishui Municipal Central Hospital, Lishui, Zhejiang Province, People's Republic of China.

**Keywords:** lateral neck mass, lymphangioma, papillary thyroid micro-carcinoma, thyroid, thyroid cancer

## Abstract

**Introduction:**

Metastasis of a papillary thyroid microcarcinoma (PTMC) in the lateral neck is characterized primarily by solid lymphadenopathy, although some cases may rarely present with a cervical cystic mass. We report a case of lateral cervical lymph node metastases of PTMC that appeared as a cystic lymphangioma of the lateral neck.

**Patient concerns:**

A 55-year-old man with a painless egg-sized mass in the right side of the neck that had been present for 1 month underwent physical examination, ultrasonography, computed tomography (CT), fine needle aspiration biopsy (FNAB), and intraoperative fast-frozen pathological examination, which indicated that the cystic masses in the neck were benign. However, the final pathology report identified the lateral neck masses as lymph node metastases of thyroid carcinoma.

**Diagnosis:**

The patient was diagnosed with PTMC of the right lobe of the thyroid gland with lateral neck metastases.

**Interventions:**

The patient underwent right cervical neck dissection together with a right thyroidectomy, followed by levothyroxine therapy and routine follow-up.

**Outcomes:**

No postoperative complications were reported, and the thyroid-stimulating hormone inhibition target was <0.1 mmol/L; there was no detectable tumor recurrence on routine clinical follow-up for up to 16 months.

**Conclusions:**

This case report emphasizes the need to consider cervical lymph node metastases of thyroid carcinoma in the differential diagnosis for patients with large, multiple, simple cystic neck masses.

## 1 Introduction

Papillary carcinoma of the thyroid is the commonest malignant tumor of the thyroid, and papillary thyroid microcarcinoma (PTMC) accounts for the majority of cases with papillary carcinoma.^[1]^ Most papillary thyroid carcinomas manifest as a solitary thyroid nodule or solid enlarged lymph nodes in the lateral neck region. However, lateral cervical metastasis can present with similar cystic changes in the lateral and middle neck regions. More than 50 case reports have been published on cystic metastasis of PTMC in the lateral neck. However, multiple, simple cystic metastasis of PTMC is extremely rare, and this case is only the third to be reported in the literature. Cystic masses of the lateral neck are difficult to diagnose preoperatively, and simple imaging features cannot be used as the basis for diagnosis.^[2]^ This report presents a case of PTMC with the initial symptom of multiple simple cystic lesions in the lateral neck.

## 2 Case report

**Figure 1:**
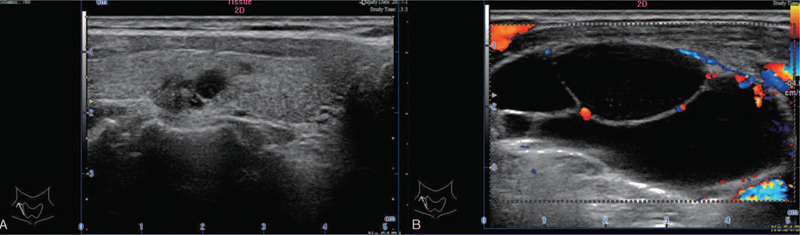
(A) The nodule of thyroid gland. (B) The size of the larger one reached 6.2 × 4.8 × 2.4 cm^3^, showing multilocular changes.

**Figure 2:**
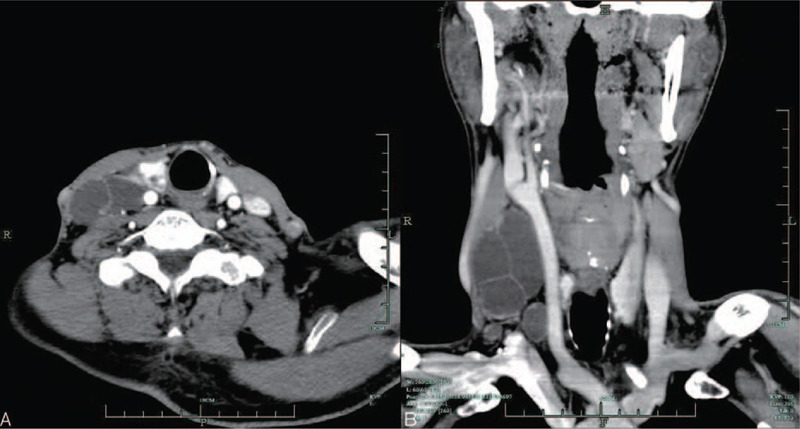
(A) There were two nodules in the thyroid gland. (B) The mass on the right side of the neck showed a well-defined cystic lesion.

A 55-year-old man presented to our department with a painless egg-sized mass in the right side of the neck that had appeared 1 month ago, but had no symptoms, such as hoarseness or dysphagia. The patient had no family history of tumors. Physical examination revealed a smooth, oval, painless mass (6 × 5 cm) in the right side of the neck. The mass was located behind the right sternocleidomastoid muscle, with many smaller small masses (1–2 cm each) in the surrounding area. Ultrasonographic imaging showed many cystic dark areas in the right side of the neck, and the largest cystic area measured 6.2 × 4.8 × 2.4 cm^3^ and displayed multilocular changes (Fig. 1B). Multiple nodules were detected in the right thyroid gland, including a hypoechoic solid nodule (0.7 cm) with irregular margins and strong echogenic dots. Therefore, the nodule of the thyroid gland was graded as TR5 based on the 2017 ACR Thyroid Imaging Reporting and Data System (ACR 2017 TI-RADS; Fig. 1A). Fine needle aspiration biopsy (FNAB) showed that the cystic masses in the neck were benign, whereas the thyroid nodule was malignant. On computed tomography (CT) scanning, there were two nodules in the thyroid gland (Fig. 2A); the masses on the right side of the neck showed a well-defined cystic lesion (Fig. 2A and B).

**Figure 3:**
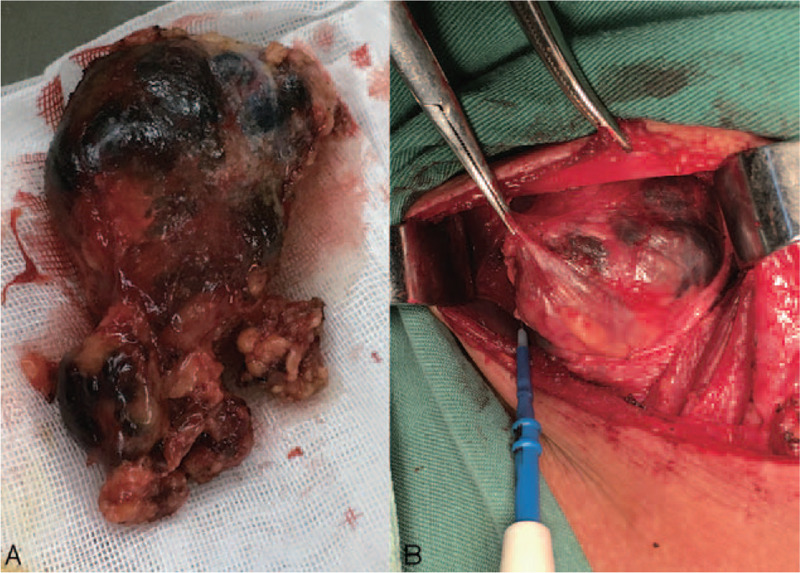
The excised cervical mass.

**Figure 4:**
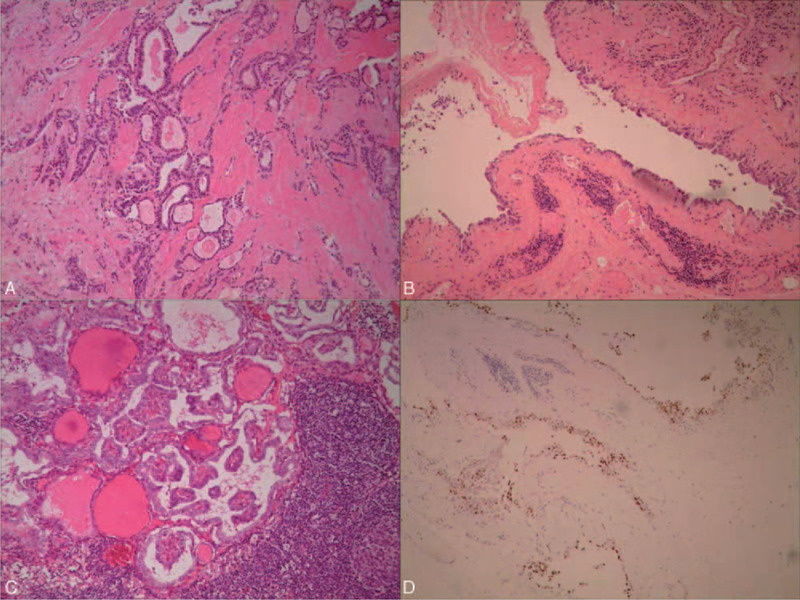
(A) A thyroid carcinoma focus, with obvious interstitial fibrosis, enlarged and elongated cancer cell nucleus, parts of which showed a nuclear groove, irregular follicular arrangement of tumor cells, and parts with papillary formation and invasive growth (100×; hematoxylin eosin [HE] staining). (B) The cystic changes within the cervical metastasis, which was internally lined with cuboidal or low columnar epithelium and had a slightly heteromorphic nucleus (100×, HE staining). (C) A papillary thyroid carcinoma with metastatic lymph nodes, with an obvious typical papillary appearance (200×, HE staining). (D) TTF1 was positive in the lined epithelium of cervical metastasis (100×; TTF1 staining).

Preoperatively, there was no evidence of malignancy in the right cervical masses; however, our clinical team speculated that the right cystic masses might possibly be malignant metastases of the thyroid tumor. Therefore, the right cervical lymph nodes were intraoperatively dissected in the patient (from areas II to V), with a parallel operation of total thyroidectomy. The excised cervical masses measured 9.2 × 6.4 × 3.5 cm^3^ (Fig. 3A and B). Intraoperative fast-frozen pathological examination showed a papillary carcinoma of the right thyroid gland and isthmus (largest diameter 0.8 cm), and the lateral cervical masses were considered to be a lymphangioma or cystic lesion. No further resection of the left thyroid gland lobe was undertaken in view of the histopathological findings. However, the routine postoperative pathological examination revealed unexpected findings. The view of the focus of the right thyroid gland (100×; hematoxylin eosin [HE] staining) showed a thyroid carcinoma focus, with obvious interstitial fibrosis with enlarged and elongated cancer cell nuclei, parts which showed a nuclear groove, irregular follicular arrangement of tumor cells, and papillary formation with invasive growth (Fig. 4A). Meanwhile, an examination of the lymph node (the largest cystic mass; 100×, HE staining) showed cystic changes within the cervical metastatic mass, which was internally lined with cuboidal or low columnar epithelium and had a slightly heteromorphic nucleus (Fig. 4B). Moreover, HE staining under a higher magnification of 200× indicated a papillary thyroid carcinoma with metastatic lymph nodes, with an obvious typical papillary appearance (Fig. 4C). Furthermore, immunohistochemical staining (100×; TTF1 staining) showed positive staining for TTF1 in the lining epithelium of the cervical metastases (Fig. 4D). The other multiple smaller cystic masses showed similar characteristics. The final pathological report indicated a papillary carcinoma of the right thyroid gland lobe and isthmus (largest diameter 0.8 cm), and that the masses in the lateral neck were lymph node metastases of the thyroid carcinoma.

The patient had no obvious complications during the perioperative period. Based on the postoperative histopathological report, the patient was advised reoperation for the resection of the left lobe of the thyroid gland, but refused surgical reintervention. Therefore, the patient was prescribed pharmacotherapy with levothyroxine for thyrotropin (thyroid stimulating hormone [TSH])-suppressive therapy to reduce the TSH to <0.1 mIU/L. At the time of this report, the patient has been followed up for ∼16 months and had no recurrence or metastasis.

## 3 Discussion

The incidence of thyroid carcinoma has more than doubled over the past three decades,^[3]^ and is mainly attributable to papillary thyroid carcinoma, which is predominantly PTMC.^[1]^ Ito et al^[4]^ did not support surgical resection for low-risk patients with PTMC. Moreover, the American Thyroid Association (ATA) Guidelines^[5]^ suggest careful observation for the management of PTMC. However, the rate of metastasis in the central region of PTMC is extremely high, reaching 24.1% to 64.1%, and this rate approaches 3.7% to 44.5% in the lateral neck,^[6]^ which suggests the possibility of PTMC invasiveness. Nonetheless, the present issue in PTMC management is that it is difficult to determine the invasiveness of papillary thyroid carcinoma.

Furthermore, in this rare case, the invasiveness of the PTMC manifested an initial symptom of a solitary cystic metastasis of the lateral neck region.^[7]^ In addition, it is extremely rare to have multiple simple cystic masses with symptoms that mimic a lymphangioma, as in this case.

A subcortical liquefying necrosis was the cause of the cystic metastasis of PTMC in the lateral neck.^[7]^ Ultrasonography is the first choice for thyroid examination, although it has a relative low sensitivity in the diagnosis of cystic masses of the lateral neck. In this case report, an ultrasound showed multi-septate cystic masses in the right side of the neck, indicating the possibility of a lymphangioma. Subsequent ultrasonographic examination of the thyroid gland showed a nodule that was suspected to be malignant, which was confirmed in the subsequent FNAB. However, FNAB of the lateral cervical cystic masses indicated benign lesions, which underscores the lack of accuracy of FNAB in the diagnosis of cystic lesions. The false-negative range of FNAB is 33% to 83% in the diagnosis of cervical cystic metastasis.^[8]^ However, this may be attributed to sampling errors, and specimens can be obtained from the wall or solid part of the capsule to improve diagnostic accuracy. Moreover, the level of thyroglobulin (TG) can be measured to evaluate a metastatic cystic lesion of the thyroid. However, we did not test the intracystic TG level with FNAB. Furthermore, CT scanning can confirm the findings of ultrasonography and determine the extent of lesions; however, CT scanning has limitations in the diagnosis of non-calcified metastasis to the lateral neck. Preoperatively, our patient was diagnosed as a cervical cystic lymphangioma, but was eventually proved to have cervical lymph node metastasis on postoperative histopathological examination. In the present case, both ultrasonography and CT scanning showed that a lymphangioma was the root cause of the cervical mass, and there was no malignant indication on the intraoperative fast-frozen pathology of the lateral cervical cyst. Consequently, the patient was treated with extensive lymph node dissection of the lateral neck, and the right lobe of the thyroid was removed intraoperatively. The patient refused to undergo a more thorough reoperation, and this may result in a higher risk of recurrence and metastasis. The patient is currently on pharmacotherapy with levothyroxine, and is closely followed up to ensure that the TSH level is suppressed to <0.1 mIU/L.^[5]^

## 4 Conclusion

Most cystic masses of the lateral neck are benign, although malignant diseases such as PTMC must be considered in the differential diagnosis. There is reason to believe that, despite the presence of clear benign lesions, the examination of cystic masses in the neck of adult patients requires a high degree of clinical suspicion. In the presence of a primary malignant lesion of the thyroid with a negative result on FNAB of a lateral cervical cystic mass, a total thyroidectomy and neck lymph node dissection in the primary resection is recommended to reduce the risk of recurrence and metastasis.

## Acknowledgments

We would like to thank Editage (www.editage.com) for English language editing.

## Author contributions

**Conceptualization:** Yong Wu, Feng Cheng.

**Formal analysis:** Xi Zhu.

**Investigation:** Bin Zhou, Yong Wu.

**Resources:** Wei bo Mao.

**Supervision:** Feng Cheng.

**Writing – original draft:** Lei Zhu.

**Writing – review & editing:** Lei Zhu.

## Abbreviations

Abbreviations: CT = computed tomography, FNAB = fine needle aspiration biopsy, PTMC = papillary thyroid micro-carcinoma.
